# Disseminated Legionella Associated With Myocarditis in an Otherwise Immunocompetent Host: A Case Report and Review of the Literature

**DOI:** 10.7759/cureus.40529

**Published:** 2023-06-16

**Authors:** Abdullah Jarrah, Mohamed Mansour, Ahmad Alnasarat, Ahmed Abdelrahman, Ahmad Damlakhy, Sherif Eltawansy

**Affiliations:** 1 Internal Medicine, Detroit Medical Center/Sinai Grace Hospital/Wayne State University, Detroit, USA; 2 Internal Medicine, Jersey Shore University Medical Center, Neptune, USA

**Keywords:** legionella pneumophila serogroup 1, legionella induced heart failure with reduced ejection fraction, legionella induced myocarditis, extrapulmonary legionella, legionella pneumonia

## Abstract

Legionnaires’ disease caused by the bacteria Legionella pneumophila, is considered a type of atypical pneumonia. The disease usually presents with dyspnea, cough, fever, muscle aches, headache, nausea, and vomiting. A milder form of the disease (Pontiac fever) with flu-like illness also exists. In addition to lung infection, extrapulmonary manifestations might occur including sepsis, rhabdomyolysis, neurological impairment, kidney, and liver damage. Myocarditis can be seen as a rare complication in Legionnaires’ disease. Here, we are presenting a case of Legionnaires’ disease associated with myocarditis in a patient with no predisposing risk factors for severe illness.

## Introduction

Legionella is considered an uncommon cause of pneumonia and is responsible for 1%-3% of community-acquired pneumonias (CAP) [1]. Humans contract the bacteria from contaminated environmental sources by inhaling aerosols or aspirating liquids [2]. The illness does not spread from person to person, and there is currently no vaccine available to prevent it [3]. Risk factors for infection include old age, chronic lung disease, smoking, immunodeficiency, and active cancer or receiving chemotherapy treatment [4]. In addition to pneumonia, extrapulmonary manifestations are rare, but well-known complications of legionella pneumonia. Myocarditis is considered a rare extrapulmonary complication of legionella pneumonia with few reported cases in the literature [5-7].

## Case presentation

A 61-year-old male patient with a history of essential hypertension presented to the emergency department with a chief complaint of cough for one week. He had a moderate amount of sputum, brown in color with no blood. He also complained of mild shortness of breath, sore throat, and runny nose. The patient also reported dark urine color but no change in frequency or burning with urination. He denied any chest pain or orthopnea. He had no associated fever, chills, or rigors.

**Figure 1:**
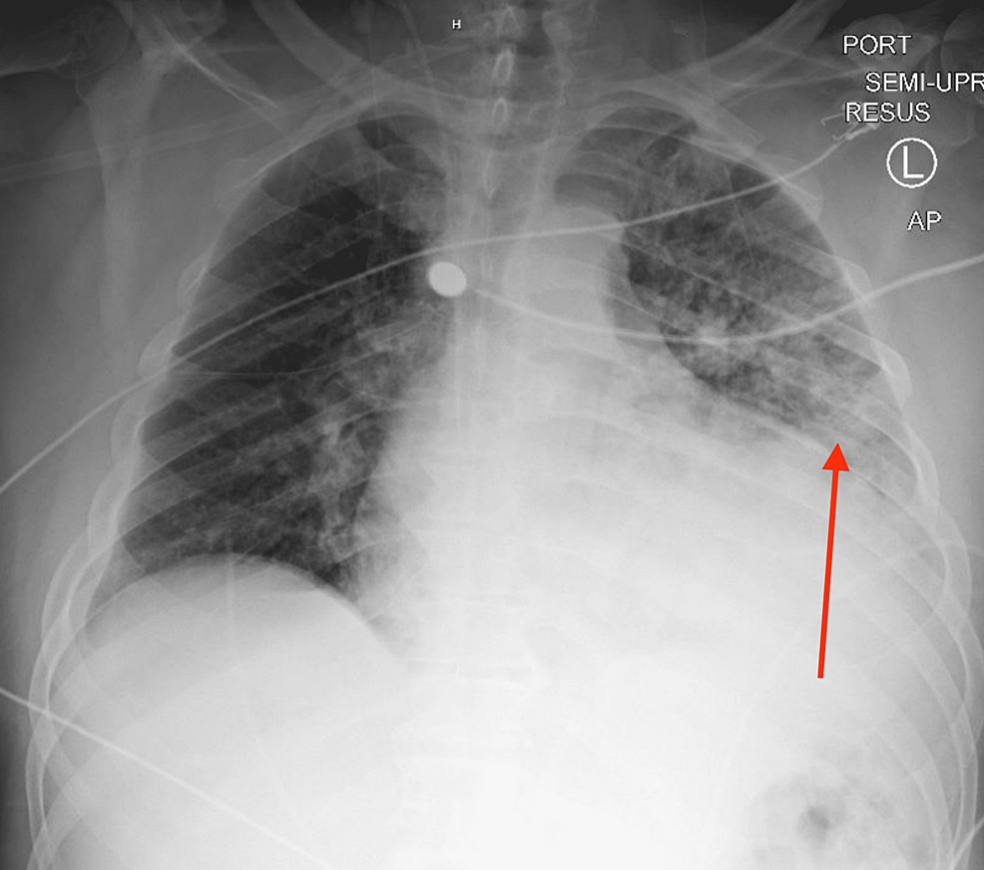
CXR showing patchy left lower lobe opacification and pulmonary congestion as pointed by the red arrow

Physical examination initially was significant for crepitation in the left lower lung, with a bilateral decrease in air entry in lung bases. Laboratory evaluation showed leukocytosis of 26.9 K/mm^3^ with 85% neutrophils, creatinine of 3.1 mg/dL, blood urea nitrogen (BUN) of 74 mg/dL, and creatine phosphokinase (CPK) level of 7,488 units/L. Also, he had elevated alanine aminotransferase (ALT) and aspartate aminotransferase (AST) of 81 units/L and 171 units/L, respectively. Brain natriuretic peptide was elevated at 1,416 pg/mL, troponin level of 361 ng/mL that was repeated two hours after with a level of 189 ng/mL. Chest x-ray (CXR) showed patchy left lower lobe opacification and consolidation, in addition to pulmonary congestion (Figure 1).

Initial vitals were blood pressure (BP) 107/73 mmHg, heart rate (HR) 91 beat per minute (BPM), respiratory rate (RR) 18 breath/minute, temperature 37.9 Celsius, and oxygen saturation 92% on room air. Shortly after admission, the patient became tachypneic and tachycardic, with the cardiac monitor showing heart rate of 180 BPM. Electrocardiogram was done and showed atrial fibrillation with rapid ventricular response. BP dropped to 78/65 mmHg and the patient was started on oxygen via nasal cannula of 4 L/minute. He was emergently cardioverted with 100 joules, HR improved to 120 BPM, BP to 200/65 mmHg, and repeated electrocardiogram showed sinus tachycardia with no ischemic changes. As the patient continued to have tachypnea with RR of 30 breath/minute on nasal cannula, he was placed on bilevel positive airway pressure.

The patient was started on ceftriaxone and doxycycline as a treatment for CAP, and laboratory evaluation for underlying causes including sputum gram stain and culture, Streptococcus and Legionella urine antigens, and blood cultures were sent.

Echocardiogram was done and showed severe global left ventricular systolic dysfunction, with an estimated ejection fraction of 25%-30%. Urine Legionella test showed positive Legionella pneumophila serogroup one antigen suggestive of Legionella pneumonia, and the patient was diagnosed with legionnaires' disease. The antibiotics were switched to azithromycin 500 mg daily for a total of 14 days and rifampin 300 mg twice daily for five days. The patient was also treated with amiodarone for his new onset atrial fibrillation. Anticoagulation was started with heparin, then changed to apixaban five mg twice daily. During hospital stay, the patient continued to improve, and he did not require oxygen after 10 days of treatment. A follow up echocardiogram after 10 days showed improvement of ejection fraction to 55% prior to discharge. Labs on discharge were white blood cells count of 14 K/mm^3^, BUN of 40 mg/dL, creatinine of 1.41 mg/dL, ALT of 77 units/L, and AST of 37 units/L.

On his two-week follow-up, the patient continued to improve and denied any shortness of breath or palpitations. Labs were back to normal, with negative troponin. Two months afterward, the patient had elective cardiac catheterization which revealed no coronary artery obstruction.

## Discussion

Pneumonia is defined as an infection of the lung parenchyma, with an estimated annual incidence of 24.8 cases per 10,000 adults in the United States (US) [8]. The disease can be classified into CAP or hospital-acquired pneumonia (HAP). It also can be classified into infectious and non-infectious pneumonia, with infectious pneumonia further classified into bacterial, viral, fungal, or parasitic.

Legionnaires' disease is a type of atypical pneumonia that is caused by the aerobic, gram-negative bacterium legionella. The first case was described in 1976 after an outbreak in Philadelphia, US [9]. The disease usually presents with lower respiratory tract symptoms including cough, sputum production, chest pain, and shortness of breath. Symptoms may also include fever, myalgia, abdominal pain, nausea, vomiting, and confusion. A milder form of the disease without pneumonia is called Pontiac fever [10].

There are more than 50 species and 70 serogroups of the Legionella genus and almost 50% have been associated with infections in humans [11]. Although different diagnostic modalities are available to diagnose legionella infections, most cases are diagnosed by urine legionella antigen [12]. Urine legionella antigen tests for Legionella pneumophila serogroup one, account for 50%-80% of legionella cases, which implies that the diagnosis can be missed in 20%-50% of the cases. If legionella is suspected after a negative urine test, other modalities of diagnosis including respiratory culture, direct fluorescent antibody, and molecular testing can be utilized [11].

The mainstay treatment of legionnaires' disease is antibiotics and supportive measures. The most commonly used antibiotics are fluoroquinolones (levofloxacin or moxifloxacin) or macrolides (preferably azithromycin) as first-line therapy [13]. Supportive treatment includes analgesics, antipyretics, oxygen supply for hypoxemic patients, and monitoring for complications which include acute respiratory failure, dehydration, hyponatremia, sepsis, shock, gastrointestinal complications like diarrhea, acute or fulminant hepatitis, rhabdomyolysis, renal failure, multiple organ failures, neurological deficits, coma, and death [9].

Myocarditis is an inflammation of the myocardium, with viral infections being the most common cause [14]. The first case of legionella pneumonia-causing myocarditis was reported in 1981 [15]. Myocarditis due to legionella infection is rare, with few reports in the literature [5-7]. Myocarditis presentation can range from mild chest discomfort, and dyspnea, to severe cardiogenic shock [16].

Although signs and symptoms, elevated cardiac enzymes, and echocardiogram findings might suggest the diagnosis of myocarditis, the gold standard method for its diagnosis is cardiac magnetic resonance (CMR) imaging [17]. A positive endomyocardial biopsy is also diagnostic. Our patient did not get CMR due to unavailability, however, the improvement of his ejection fraction upon discharge (10 days after the initial echocardiogram) suggested that the cause was related to legionella myocarditis. Furthermore, his normal cardiac catheterization excluded the possibility of ischemic cardiomyopathy.

An important differential diagnosis in our case is stress-induced cardiomyopathy (SIC), which can present with transient left ventricular dysfunction. SIC is a clinical syndrome characterized by transient systolic heart failure, usually occurring after emotional stress. It typically occurs in postmenopausal women but can affect individuals of any age. What makes myocarditis a more likely etiology in our case is the patient's sex and the lack of typical characteristic echocardiogram findings of apical ballooning. Although CMR was not performed in our case, it is helpful in differentiation as it shows patchy late gadolinium enhancement (LGE) in myocarditis but not in SIC. However, LGE can sometimes be present in SIC as well [18].

## Conclusions

Legionella bacterium is an uncommon cause of pneumonia, which can be complicated by extrapulmonary manifestations. Myocarditis caused by legionella infection is a rare manifestation associated with Legionnaires’ disease. Although the treatment for myocarditis due to legionella infection is supportive until the underlying bacterial infection is eradicated, it is important to identify the condition to anticipate complications, counsel patients, and provide appropriate follow-up.
